# Half-Dose Anticoagulation for Venous Thromboembolism and Phlegmasia Cerulea Dolens in a Patient With Recurrent Subdural Hematoma

**DOI:** 10.7759/cureus.18048

**Published:** 2021-09-17

**Authors:** Junfei Hu, Kelley Chan, Nirajan Adhikari, Amber Khan

**Affiliations:** 1 Internal Medicine, Guthrie Robert Packer Hospital, Sayre, USA; 2 Internal Medicine, Geisinger Commonwealth School of Medicine, Scranton, USA

**Keywords:** half-dose anticoagulation, case report, subdural hematoma, phlegmasia cerulea dolens, venous thromboembolism

## Abstract

Anticoagulation for venous thromboembolism (VTE) in patients with recurrent subdural hematoma (SDH) is challenging. It becomes even more challenging when the patient develops phlegmasia cerulea dolens (PCD). We present a 66-year-old female with a recent history of recurrent SDH who received half-dose heparin therapy for VTE and PCD. The patient had improvement of dyspnea and resolution of PCD after two days of treatment. She was discharged with half-dose enoxaparin. At her one-month follow-up, there was no evidence of new SDH or progression of VTE. Half-dose anticoagulation therapy should be considered in patients with recurrent SDH when anticoagulation is inevitable.

## Introduction

Intracranial hemorrhage (ICH) predisposes patients to the development of venous thromboembolism (VTE) due to immobility and hypercoagulability [1]. In patients with recent ICH who develop VTE, anticoagulation is contraindicated, and inferior vena cava (IVC) filter is recommended. We report a case of a 66-year-old female with recent recurrent subdural hematoma (SDH) who developed progressive VTE and phlegmasia cerulea dolens (PCD) after an IVC placement. Data were limited in regards to initiation of anticoagulation in the setting of recent SDH. We proceeded with treatment with half-dose anticoagulation therapy for VTE and PCD.

## Case presentation

**Figure 1:**
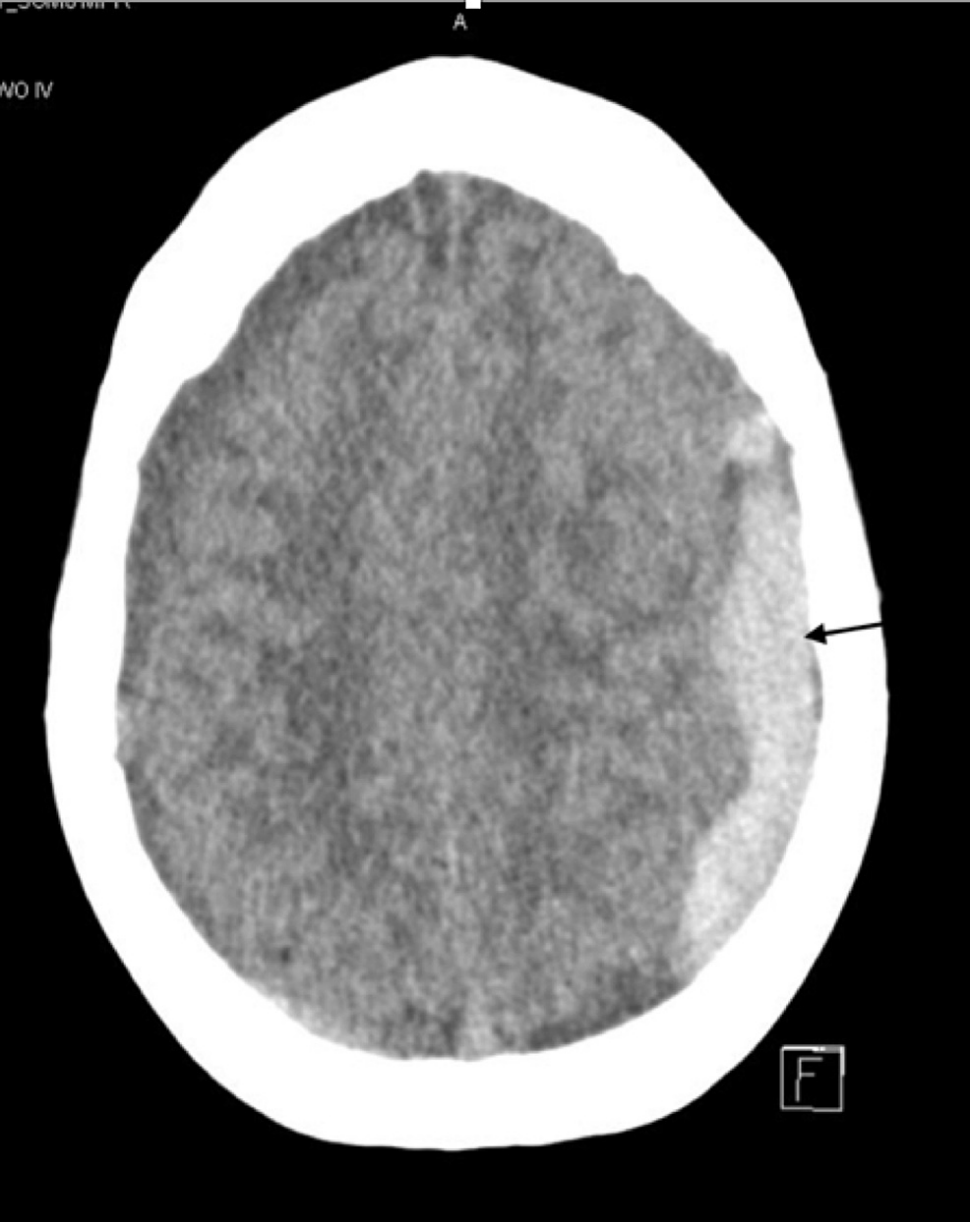
Brain computed tomography. Longitudinal section: left subdural hematoma.

**Figure 2:**
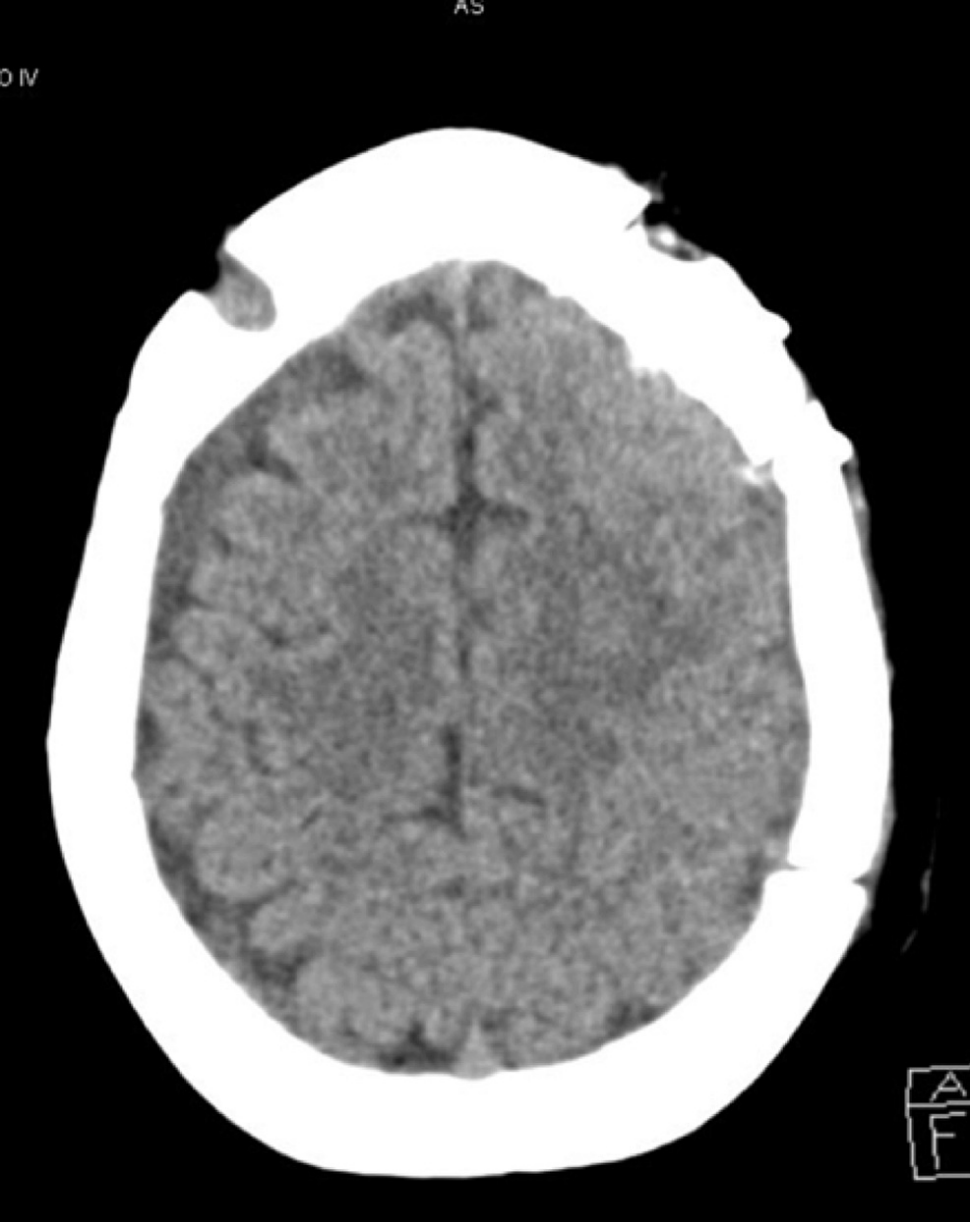
Brain CT. Longitudinal section: resolution of left subdural hematoma after burr hole surgery.

**Figure 3:**
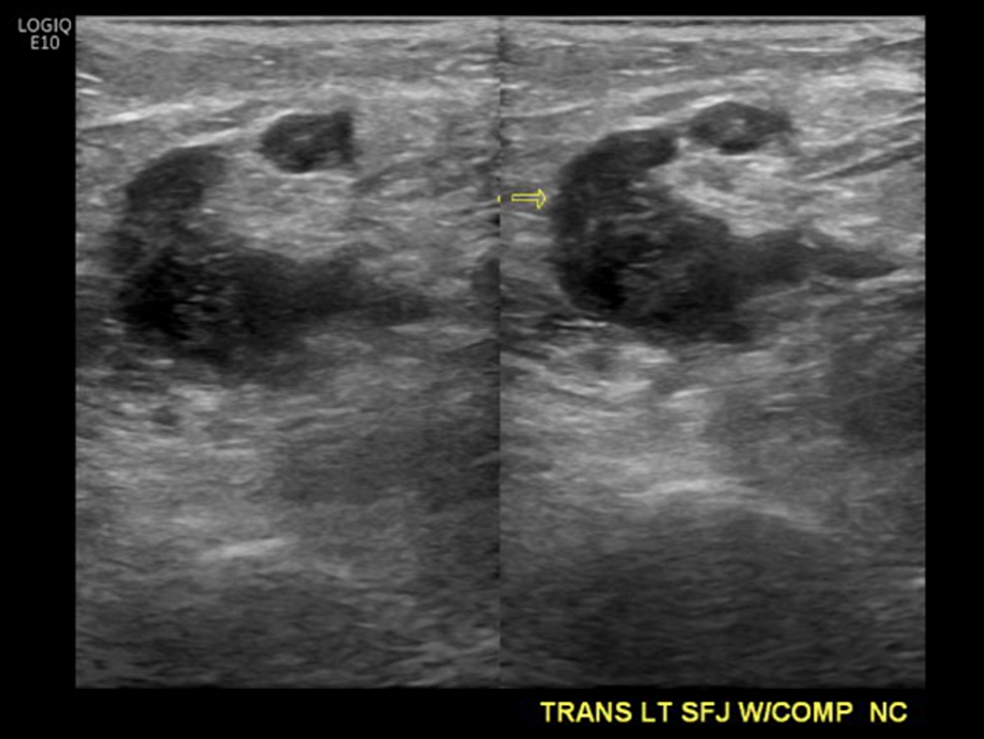
Lower extremity venous Doppler. Left non-occlusive DVT in saphenofemoral junction. DVT, deep venous thrombosis.

**Figure 4:**
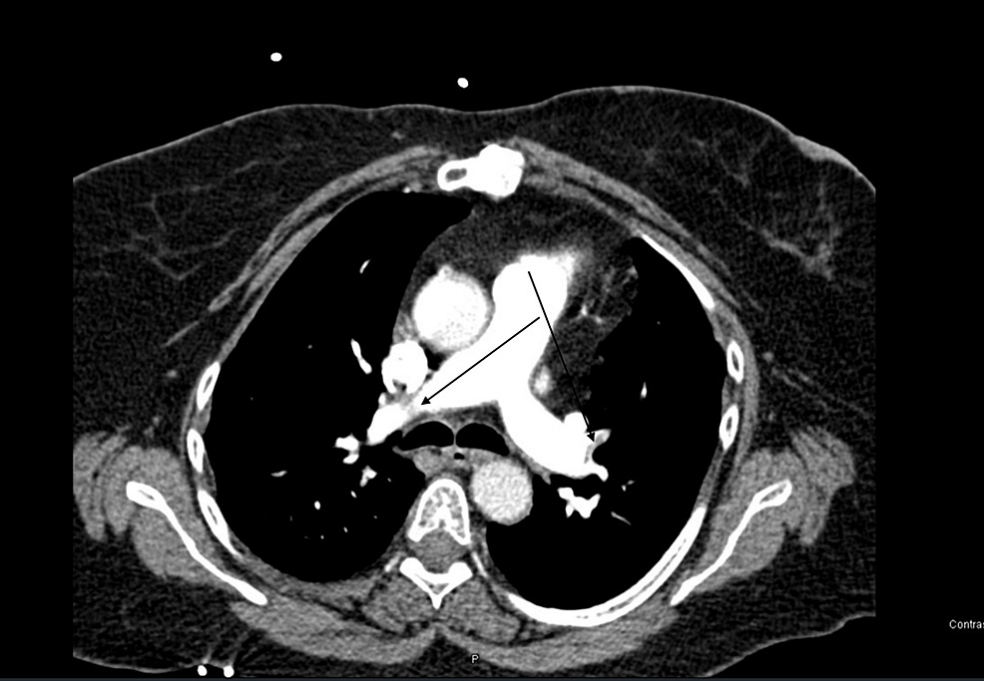
CT PE. Bilateral PE. PE, pulmonary embolism.

A 66-year-old female with a past medical history of VTE, aortic stenosis (AS) status post mechanical aortic valve replacement (AVR) on warfarin, and recent SDH presented to the emergency department (ED) for new-onset shortness of breath (SOB) and ongoing left leg pain. Two months prior, the patient developed a spontaneous SDH while on warfarin (Figure 1). Neurosurgery performed bilateral burr holes for evacuation of the hematoma (Figure 2); however, she still subsequently developed recurrent SDH that required three craniotomies and evacuations over the next month. Her warfarin was held after the first recurrence of SDH. She then developed left lower extremity pain and swelling. At the time, the lower extremity Doppler showed an acute non-occlusive deep venous thrombosis (DVT) in the left proximal femoral vein, popliteal vein, and calf vessels (Figure 3). Anticoagulation was contraindicated; thus, an IVC filter was placed. Two days later, she developed SOB and presented to the ED. Her vital signs were stable. CT pulmonary embolism (PE) showed multiple bilateral PE (Figure 4). She had asymmetrical left leg edema and tenderness. The lower extremity Doppler of the left leg showed an acute occlusive DVT extending from the posterior tibial and peroneal veins up to the inferior vena cava (IVC), which had worsened when compared to the previous Doppler obtained two days prior.

**Figure 5:**
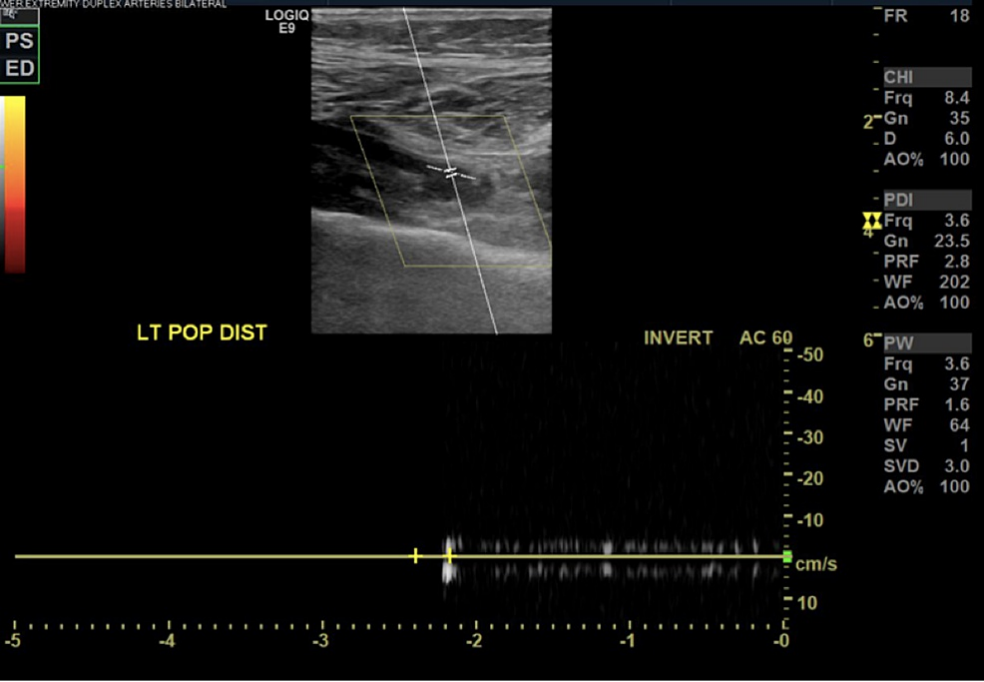
Lower extremity arterial Doppler. No flow in the left distal popliteal artery.

**Figure 6:**
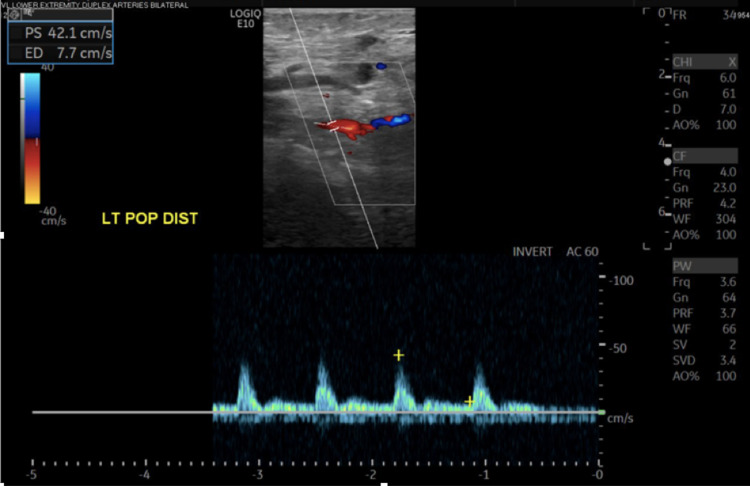
Lower extremity Doppler. Improved blood flow in the left distal popliteal artery after heparin treatment.

A multidisciplinary approach among internal medicine, neurosurgery, and hematology regarding initiation of anticoagulation therapy was held; however, the patient started to have intractable left lower extremity pain that could not be controlled with high-dose opioids. Her left leg was pale and edematous up to the thigh. Ankle-brachial index (ABI) was 0.64 on the left side, indicating moderate peripheral arterial disease (PAD), and lower extremity arterial Doppler showed occlusion of the left popliteal artery (Figure 5). She was thought to have developed phlegmasia cerulea dolens (PCD) in the setting of extensive DVT and recent IVC filter placement. Opinions were obtained from cardiology and interventional radiology in regards to thrombectomy versus thrombolysis. The patient was not medically optimized for these procedures. After discussion with neurosurgery, hematology, and vascular surgery, the patient was started on half-dose anticoagulation therapy. Half-dose heparin was initiated with a goal of partial thromboplastin time (PTT) between 45 and 50. The patient's left leg pain resolved after two days of half-dose heparin treatment. Repeat ABI was 0.69, and the lower extremity arterial Doppler showed reopening of the left popliteal artery (Figure 6).

Two days after the initiation of half-dose heparin, the patient had one episode of rectal bleeding and dark emesis. The heparin drip was discontinued for concerns of gastrointestinal bleeding. Her hospitalization was complicated by ileus and acute kidney injury (AKI) from contrast-induced nephropathy. General surgery and gastroenterology were consulted for ileus. A nasogastric tube was placed for decompression and her ileus was resolved with conservative management. Nephrology was consulted to manage her AKI. Her creatinine peaked at 3.4 mg/dL and slowly improved after diuresis and supportive care. Three days after the heparin drip was discontinued, her hemoglobin remained stable, and there was no recurrence of bleeding. The decision was made to restart her on half-dose heparin. She continued to improve and was discharged home with half-dose enoxaparin. At her one-month follow-up, there was no evidence of new SDH or progression of VTE. Her renal function returned to baseline, and she was tolerating oral intake. She will remain on half-dose enoxaparin until cleared by hematology and neurosurgery to switch her back to warfarin.

## Discussion

Patients with ICH have an increased risk of developing VTE. Immobility and activation of the coagulation cascade after an ICH are the two significant factors predisposing patients to VTE [1]. After recent ICH, holding anticoagulation increases the risk of DVT to 2% to 15% and PE to 1% to 5% [2-3]. Given the high risk of VTE, these patients may require anticoagulation therapy despite the risk of recurrent ICH. A review article found that while the resumption of anticoagulation in patients with recent ICH decreases the risk of VTE, there are conflicting data regarding the risk of recurrent ICH [4].

In our case, anticoagulation was deemed necessary, despite the high risk of recurrent SDH, as the progression of VTE would have put the patient’s life at risk. Furthermore, the patient also developed PCD, which made anticoagulation imperative, especially since the surgical intervention was contraindicated. PCD is a rare condition that occurs when extensive DVT causes occlusion of venous return and subsequent arterial occlusion due to elevated compartment pressure from fluid retention. This condition is more common on the left side and in patients in their 50s and 60s. Additionally, recent IVC filter placement is a risk factor for PCD [5]. Most experts recommend treating PCD with thrombolysis, with heparin as the preferred agent, or thrombectomy [6]. One case reported the successful treatment of PCD with apixaban in a patient with contraindications for surgical intervention [7]. Although thrombolysis was contraindicated in our patient due to recent recurrent SDH, her condition was not stable enough for thrombectomy. Thus, we started her on half-dose heparin.

A literature review of the efficacy and safety of current major anticoagulation medications was conducted. A case report using novel oral anticoagulants (NOAC) for PE treatment after recent ICH showed that patients did not have a recurrence of ICH or progression of VTE at their three-week and one-year follow-up [8]. A study comparing the outcomes using direct-acting oral anticoagulants (DOAC) or warfarin for VTE in patients with ICH showed that DOACs were less likely to cause ICH than warfarin [9]. Unfortunately, no data are available for evaluating the efficacy and safety of using heparin or enoxaparin to treat VTE in patients with recent ICH. We opted to use heparin and enoxaparin because they have consistent effects compared to warfarin, which can have variable effects that make it difficult to maintain the therapeutic international normalized ratio (INR) range. Additionally, heparin and enoxaparin can be easily initiated and discontinued for procedures or minor bleeding and have a quicker onset and offset compared to NOACs or DOACs.

Anticoagulation dosage must be guided to balance the risk of VTE and recurrent ICH. A study showed half-dose rivaroxaban to be as effective as full-dose rivaroxaban in treating VTE, with no significant difference in the development of recurrent VTE or bleeding events [10]. Another study using half-dose enoxaparin for VTE in cancer patients, for less than one-month duration, showed no recurrence of VTE or major bleeding events when the anticoagulant dose was reduced or held [11]. There are no studies comparing the efficacy and safety of using half-dose heparin in treating VTE. It is up to the clinician to decide the optimal therapeutic PTT goal for specific situations.

## Conclusions

Half-dose anticoagulation can be considered in patients with acute VTE and PCD when full-dose anticoagulation is contraindicated. Additional clinical studies evaluating the efficacy and risks of half-dose anticoagulation may guide VTE treatment in patients with recurrent SDH.
